# Medically Refractory Neuroborreliosis Case Presented with Coexistance Involvements of Cranial 7 and 8 Nerves

**DOI:** 10.3390/neurolint13010012

**Published:** 2021-03-18

**Authors:** Anam Hareem, Iman Dabiri, Nida Zaheer, Ahmet Z. Burakgazi

**Affiliations:** 1 Virginia Tech Carilion School of Medicine, Roanoke, VA 24016, USA; ahareem@carilionclinic.org (A.H.); idabiri@carilionclinic.org (I.D.); 2 Lahore Medical and Dental College, Lahore 53400, Pakistan; drnidazaheer@gmail.com

**Keywords:** neuroborreliosis, ixodes, facial palsy, treatment resistance

## Abstract

In the US, Lyme disease (LD) has become the most common vector-borne disease. Less than 10% of patients develop cranial nerve palsy or meningitis. There are few reports on cases of Lyme disease with more than one cranial neuropathy. Herein, we will discuss a case of persistent neurological deficits as a result of chronic Lyme disease resistant to standard therapy. Our case is unique due to involvements of cranial seven and eight nerves at the same time. Our case illustrates an extreme example of treatment resistance. However, early diagnosis and prompt establishment of adequate antibiotic treatment are still important to prevent progression to further stages of disease.

## 1. Introduction

In the US, Lyme disease (LD) has become the most common vector-borne disease [1,2]. It is transmitted by the bite of the ixodes tick, primarily ixodes scapularis (deer tick). These ticks transmit spirochetes to humans but typically require about 24–48 h of attachment [3,4]. The *spirochete borrelia burgdorferi* is the most common cause of Lyme disease. In the US, approximately 30,000 cases are reported to the Center for Disease Control (CDC) every year. The disease is endemic in the following states: Connecticut, Delaware, Maryland, Massachusetts, Minnesota, New Jersey, New York, Pennsylvania, Rhode Island, and Wisconsin [2,4].

Neurological manifestations of LD, first described by Garin and Bujadoux in 1922, have been seen to occur isolated in 12% of acute LD cases and may present as early as 2–18 weeks after exposure [2,5]. Central nervous system (CNS), as well as peripheral nervous system (PNS), manifestations can occur in isolation or together [4,5,6,7,8]. PNS involvement of cranial or peripheral nerves is the more common neurological finding and occurs in roughly 10% of infected untreated patients [4,6,7,9,10]. Radiculitis or inflammation of the nerve root can be seen 3–5% of the time in acute neuroborreliosis, affecting the PNS with a typical presentation involving intractable pain, as well as muscle denervation and areflexia over one or a few adjacent dermatomes [11]. Meningitis affecting the CNS is usually seen 1% of the time, these cases may present variable symptoms, and in rare cases, patients may develop brain parenchyma or spinal cord inflammation [4,5,9,12,13,14]. Herein, we will discuss a case of persistent neurological deficits as a result of chronic LD resistant to standard therapy.

## 2. Case Presentation

We present the case of a seventy-seven year-old male with a past medical history of hypertension and a remote history of prostate cancer treated with radiation therapy, hyperlipidemia, and coronary artery disease, who presented to his primary care physician (PCP) in August 2017 with ongoing symptoms of sore throat, right-sided ear pain, headache, and dizziness. This was associated with nausea, neck pain, and tinnitus. He was seen in-office a week earlier with upper respiratory tract symptoms and was prescribed dexamethasone. He returned for re-evaluation due to minimal improvement of symptoms and emergence of new bothersome symptoms. He did report history of a tick bite in 2016 but denies any recent tick. He denies any preceding fever, skin rash, syncopal episode, disequilibrium or further neurological deficits. On examination, he had normal vital signs and a slight asymmetry of his smile was noticed by his PCP, which prompted investigation for LD. ELISA test was positive for LD. A CT scan was also ordered to rule out any intracranial pathology and was found unremarkable.

**Figure 1:**
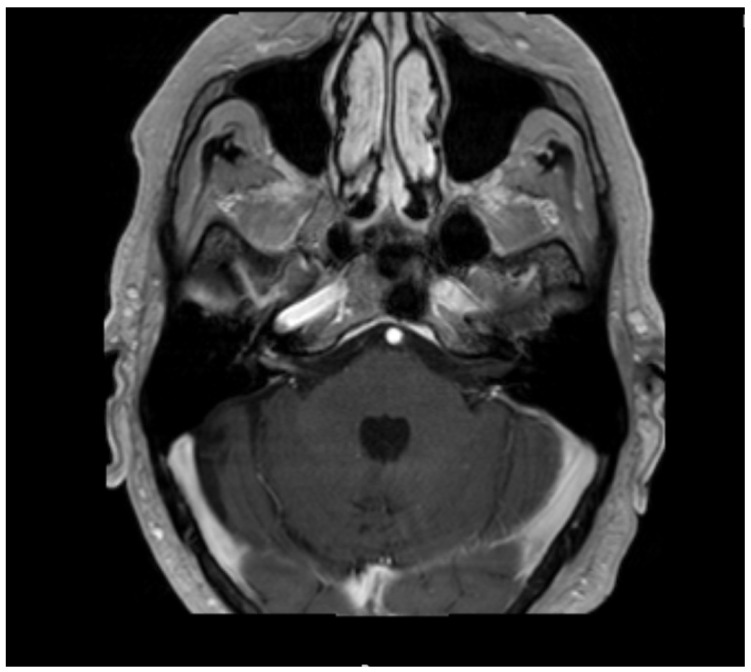
The MRI of brain showed linear enhancement of the 7th and 8th cranial nerves (CN) within the internal auditory canal, which is indicative of inflammation of the two cranial nerves.

He was subsequently started on a one-week course of doxycycline for LD. On his next visit, he complained of continuing right-sided headache and neck pain along with right-sided hearing loss, right-sided otalgia, dizziness, and nausea. There was also an examination that was suggestive of complete facial palsy. Patient was started on cefuroxime and his doxycycline was discontinued. After completing a two-week course of cefuroxime, he continued to complain of gait instability and otalgia with hearing loss and neck pain. There was no significant improvement in his facial palsy, and he received a diagnosis of disseminated LD. He was prescribed another thirty-day course of doxycycline with a referral to a neurologist who believed that the etiology of his unilateral right-sided hearing loss was related to shingles. He was also referred to physical therapy for balance training. An MRI of the brain with and without contrast was also ordered at this point. (Figure 1).

He was finally seen by neurology in May 2019 for persistent facial palsy and hearing loss with imbalance. Review of his medical records revealed an audiology evaluation indicating 80% hearing loss in the right ear and 25% in the left ear. On examination, it was noted that he had right-sided complete facial palsy and an unsteady gait. Blood work for LD Western blot with bands came back positive for active infection. His Lyme IgG immunoblot showed reactivity to six borrelial proteins and his IgM immunoblot was reactive for two proteins. He was diagnosed with chronic Lyme disease from failed antibiotic therapy with simultaneous unilateral involvement of the seventh and eighth cranial nerves. He was prescribed another doxycycline course for four weeks and showed no improvement on the subsequent follow-up visit.

## 3. Discussion

LD occurs in three stages with different clinical presentations reflecting the immune response to *borrelia burgdorferi* [4,5]. Stage one is characterized by erythema migrans (EM), fever, malaise and joint pains. EM is the characteristic rash of Lyme disease with bullseye/targetoid appearance. EM progresses from reddish papule/macule to a large erythematous annular area up to 90 cm in diameter. EM is also seen to have some atypical appearances. In the US, less than 38% of patients with EM have central clearing. Less commonly, the center may be necrotic or vesicular. EM arises within the first few weeks at the site of the tick bite in 70% of patients and often resolves after initial oral antibiotic therapy (doxycycline or amoxicillin) [10,12,15,16,17]. If the disease remains untreated, it can disseminate within months to distant organs from the original site of the tick bite.

Stage two affects the heart, skin, and nervous system. Up to 4% of patients have cardiac involvement, usually a transient atrioventricular block. Less than 10% of patients develop cranial nerve palsy or meningitis. There are few reports on cases of Lyme disease with more than one cranial neuropathy. Our case is unique, with two separate cranial nerves involved, namely seven and eight. In most cases of Lyme disease that are reported with cranial nerve involvement, the facial nerve (cranial nerve seven) is the one that is likely affected. Furthermore, an estimated 48% of patients with late stage Lyme disease may develop hearing problems, as seen in this patient [9,12,15].

MRI may provide indirect confirmation evidence of inflammation by showing enhancement of cranial nerves in patients with cranial neuropathies. In this case, MRI showed enhancements of CN7 and 8.

Stage three occurs months to years after the original exposure and exhibits neurologic defects progressing into encephalopathy, peripheral neuropathy, and arthritis [5,6,18,19]. Chronic arthritis typically involves large joints and occurs in approximately 10.8% of untreated patients. Synovial fluid from the inflamed joints raises white blood cell count, predominantly neutrophils.

The extent of case investigations varies by state. LD, frequently referred to as the “great imitator”, may resemble rheumatoid arthritis, fibromyalgia, amyotrophic lateral sclerosis, Alzheimer’s disease, multiple sclerosis, and various other autoimmune conditions. However, LD is a rare cause of death in the U.S. According to the CDC, up to five percent of untreated people have chronic neurological complaints, such as shooting pain or numbness, or memory and concentration problems, months to years later. Therefore, correct diagnosis and prompt management is important to prevent disease progression [9,20]. Various studies conducted in Massachusetts and New York showed that patients had residual symptoms of arthralgia and neurological involvement three–six years after treatment. This is known as post-treatment Lyme disease syndrome (PTLDS) [2,3,5,13]. However, the lack of any additional tests to rule out the eradication of an infection allows for an argument in support of the appropriate diagnosis being chronic LD [4,5,12].

According to the CDC, current recommendations for testing include a sensitive enzyme immunoassay (EIA) or immunofluorescence assay, followed by a Western immunoblot assay for specimens yielding positive or equivocal results. In 2019, the Food and Drug Administration (FDA) cleared several Lyme disease serologic assays with new indications for use, allowing for an EIA rather than a Western immunoblot assay as the second test in a Lyme disease testing algorithm [2].

*B. burgdorferi* has various immunogenic lipids, proteins, lipoproteins, and carbohydrate antigens. These antigens serve as potential targets for immunologic and serologic testing. The outer surface proteins OspA to -G, the 41-kDa flagellin protein, and a number of heat shock proteins are of utmost importance. The 23-kDa OspC lipoprotein is a highly immunogenic antigen and undergoes variation. Significant differences are present between the three genospecies despite expressing generally similar antigens. These differences complicate the process of having a definite serologic response [4,10,21].

An IgM immunoblot result is considered positive if any two of the three bands (the 23-, 39-, and 41-kDa bands, with the 23-kDa band representing OspC) are present. An IgG immunoblot result is considered positive if any five of the 10 bands (the 18-, 23-, 28-, 30-, 39-, 41-, 45-, 58-, 66-, and 93-kDa bands, with the 23-kDa band representing OspC) are present [2]. Western blot shows abnormal IgM and IgG antibodies. Serologic testing is insensitive in the early acute stage of Lyme disease. Furthermore, IgG antibodies become detectable after six weeks of the onset of illness and rise to a higher concentration than IgM. This provides evidence of a chronic infection. Therefore, the CDC recommendation involves detection of IgM antibodies during early weeks. ELISA can also be used for anti-borrelia antibodies, although it is non-specific [2,4,22].

Treatment with oral antibiotics is appropriate for LD when meningitis is not suspected [4,23]. In case of neurological involvement, intravenous antibiotics are recommended for two–three weeks. It is important to educate patients that antibiotics will likely treat the infection adequately, but the cranial nerve deficits may persist for weeks to months and can even be permanent.

According to the Lyme and Tick-Borne Diseases Research Center at Columbia University, “The three first-line oral antibiotics for LD include doxycycline (Monodox, Doryx, Vibramycin, Oracea), amoxicillin (Amoxil), and cefuroxime (Ceftin, Zinacef). Ceftriaxone (“Rocephin”) administered intravenously is the preferred antibiotic for neurologic Lyme disease in the United States.” [2,4,16,17,24,25].

Approximately 15% of patients treated for LD with a two-week course of antibiotics have lingering fatigue, pains, and lethargy [13]. In certain cases (as in this patient), there is no significant improvement seen after initial antibiotic therapy. For such persistent forms of these bacteria, drug combinations have been used in the past. According to various studies that have been conducted, daptomycin-containing drug combinations were the most effective. Daptomycin together with doxycycline and cefoperazone have eradicated the most resistant forms of *B. burgdorferi*. They did not yield viable spirochetes upon subculturing, and this was not achieved by any other drug combinations. These findings show implications for improved treatment options against Lyme disease [22,23].

## 4. Conclusions

Our case illustrates an extreme example of treatment resistance. Corticosteroids can also be used, but there is no significant evidence to support their efficacy in improving outcomes of cranial nerve deficits associated with Lyme disease. Early diagnosis and prompt establishment of adequate antibiotic treatment are important to prevent progression to further stages of disease.

## Data Availability

No new data were created or analyzed in this study. Data sharing is not applicable to this article.
